# Monogenic Epilepsies: Disease Mechanisms, Clinical Phenotypes, and Targeted Therapies

**DOI:** 10.1212/WNL.0000000000012744

**Published:** 2021-10-26

**Authors:** Renzo Guerrini, Simona Balestrini, Elaine C. Wirrell, Matthew C. Walker

**Affiliations:** From the Neuroscience Department (R.G., S.B.), Meyer Children's Hospital–University of Florence, Italy; Department of Clinical and Experimental Epilepsy (S.B., M.C.W.), UCL Queen Square Institute of Neurology, London; Chalfont Centre for Epilepsy (S.B.), Buckinghamshire, UK; and Divisions of Child and Adolescent Neurology and Epilepsy (E.C.W.), Department of Neurology, Mayo Clinic, Rochester, MN.

## Abstract

A monogenic etiology can be identified in up to 40% of people with severe epilepsy. To address earlier and more appropriate treatment strategies, clinicians are required to know the implications that specific genetic causes might have on pathophysiology, natural history, comorbidities, and treatment choices. In this narrative review, we summarize concepts on the genetic epilepsies based on the underlying pathophysiologic mechanisms and present the current knowledge on treatment options based on evidence provided by controlled trials or studies with lower classification of evidence. Overall, evidence robust enough to guide antiseizure medication (ASM) choices in genetic epilepsies remains limited to the more frequent conditions for which controlled trials and observational studies have been possible. Most monogenic disorders are very rare and ASM choices for them are still based on inferences drawn from observational studies and early, often anecdotal, experiences with precision therapies. Precision medicine remains applicable to only a narrow number of patients with monogenic epilepsies and may target only part of the actual functional defects. Phenotypic heterogeneity is remarkable, and some genetic mutations activate epileptogenesis through their developmental effects, which may not be reversed postnatally. Other genes seem to have pure functional consequences on excitability, acting through either loss- or gain-of-function effects, and these may have opposite treatment implications. In addition, the functional consequences of missense mutations may be difficult to predict, making precision treatment approaches considerably more complex than estimated by deterministic interpretations. Knowledge of genetic etiologies can influence the approach to surgical treatment of focal epilepsies. Identification of germline mutations in specific genes contraindicates surgery while mutations in other genes do not. Identification, quantification, and functional characterization of specific somatic mutations before surgery using CSF liquid biopsy or after surgery in brain specimens will likely be integrated in planning surgical strategies and reintervention after a first unsuccessful surgery as initial evidence suggests that mutational load may correlate with the epileptogenic zone. Promising future directions include gene manipulation by DNA or mRNA targeting; although most are still far from clinical use, some are in early phase clinical development.

An increasing number of genetic mutations and genomic rearrangements are being causally associated with a burgeoning spectrum of clinical conditions in which epilepsy is a major feature. A prospective national epidemiologic cohort study conducted in the United Kingdom estimated an overall annual minimum incidence of monogenic epilepsies in children of about 1 per 2,000 live births, with 8 genes accounting for the majority of cases.^1^ These epidemiologic figures are reflected in the detection rate of potentially pathogenic variants in up to 40% of people with different types of epilepsy, now permitted by next-generation sequencing techniques.^2,3^

The choice of antiseizure medication (ASM) in clinical practice has benefited from accumulated experience and drug trials in specific genetic conditions and from increased knowledge of the underlying disease mechanisms. Precision medicine, according to the definition promulgated by the NIH, refers to a treatment and prevention approach based on the understanding of individual variability in genetic architecture, environment, and lifestyle. One of the basic assumptions of precision medicine is that the genetic abnormality causes the phenotype, as determined through established frameworks including gene validity and variant pathogenicity. When applied to epilepsy, precision medicine may therefore include treatments addressing seizures (ASM), epileptogenesis (disease-modifying treatments), and comorbidities. However, precision medicine is currently applicable to a narrow number of patients with epilepsy and may target only part of the actual functional defects without reversing their consequences on brain development.^4^ Most monogenic disorders are rare and high-quality evidence robust enough to inform management strategies remains limited to the more frequent conditions for which controlled trials and observational studies are possible. Model systems and novel bioinformatics approaches have also been used to guide mechanistic and functional understanding of disease mechanisms, and high-throughput functional assays have been implemented for compound screening and profiling of targeted therapies. However, for the majority of rare disorders, treatment choices rely on the hypothesized functional defect or remain confined to symptom relief and general principles of epilepsy management.

In this review, we illustrate the main epilepsy phenotypes associated with monogenic disorders and the currently available treatment strategies in the most prevalent childhood-onset genetic epilepsies based on their epidemiologic framework.^1^ We also analyze the role of genetic findings in epilepsy surgery and the principles and pitfalls of gene therapy based on the established pathophysiologic mechanisms. Other important related areas such as deep phenotypic characterization, diagnostic pathways, genetic counselling, natural history, and treatment of comorbidities are beyond the scope of this review.

## Methods

We searched on PubMed for articles published from inception to January 15, 2021. We conducted our article search and selection in 2 steps. Firstly, we searched for the most prevalent epilepsy genes based on the epidemiologic framework provided in a recent study,^1^ and any related treatment options. Secondly, we conducted a broader search for less prevalent genetic epilepsies screening for all the genes listed in Tables 1–3 and any related treatment options. The following search terms were used: “seizure + specific gene name + treatment,” “epilepsy + specific gene name + treatment,” “specific gene name + treatment,” “specific gene name + precision medicine,” “gene name + antiseizure,” “gene name + epilepsy + trial.” A similar search with the specific gene names was conducted in the clinicaltrials.gov website for active treatment trials.^5^ Original research articles published in the last 5 years, that is, between 1 January 2016 and 6 January 2021, were prioritized, and older articles were selected only if including nonredundant information and treatment options still relevant in the field. Reviews were excluded. Articles were also identified through searches of the authors' own files. The search was restricted to articles published in English. The final reference list was generated based on relevance to the scope of this review.

**Table 1:**
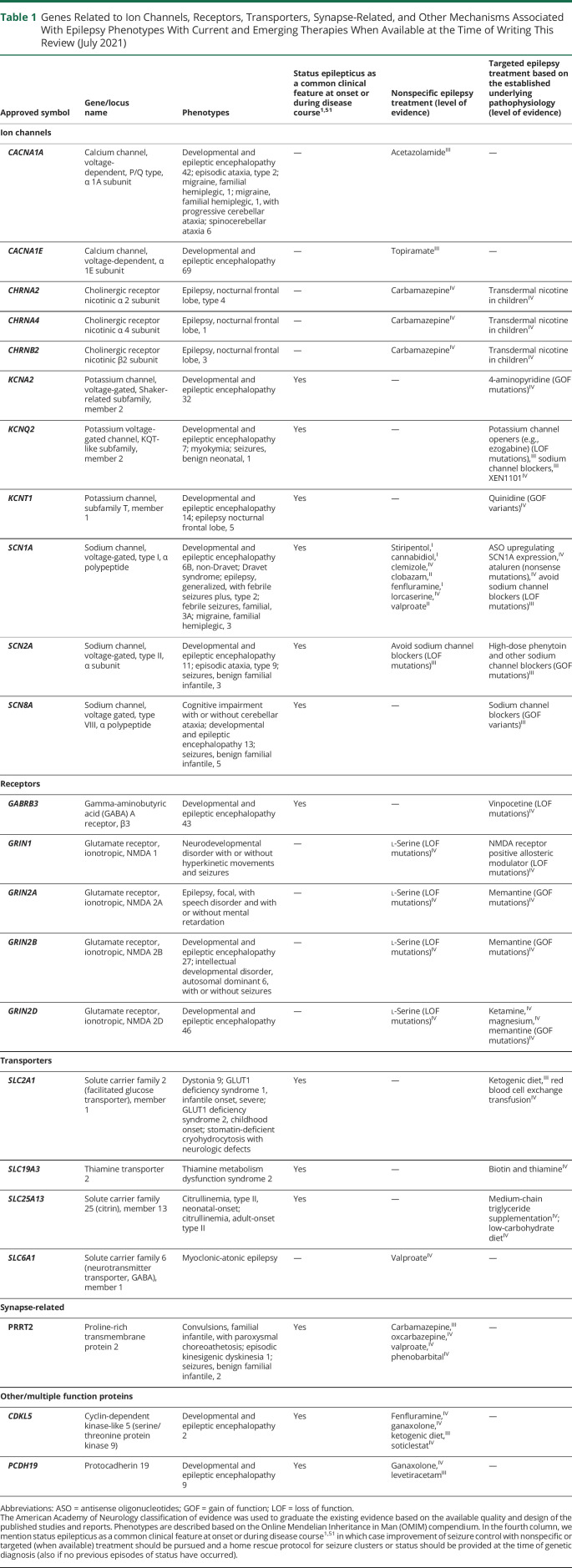
Genes Related to Ion Channels, Receptors, Transporters, Synapse-Related, and Other Mechanisms Associated With Epilepsy Phenotypes With Current and Emerging Therapies When Available at the Time of Writing This Review (July 2021)

**Table 2:**
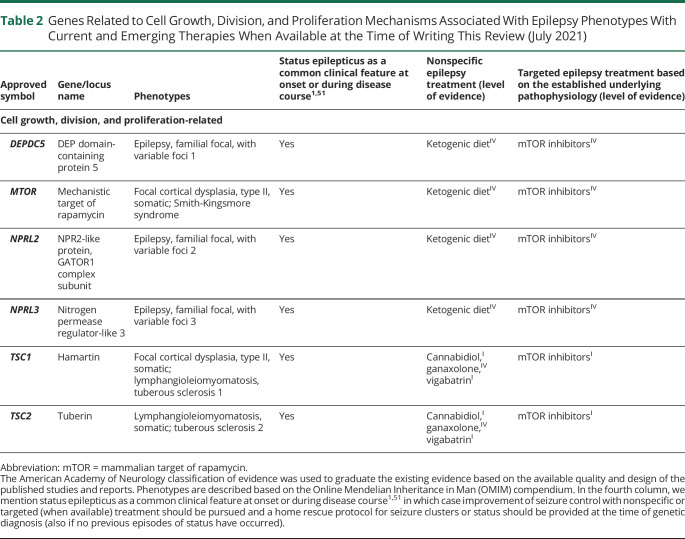
Genes Related to Cell Growth, Division, and Proliferation Mechanisms Associated With Epilepsy Phenotypes With Current and Emerging Therapies When Available at the Time of Writing This Review (July 2021)

**Table 3:**
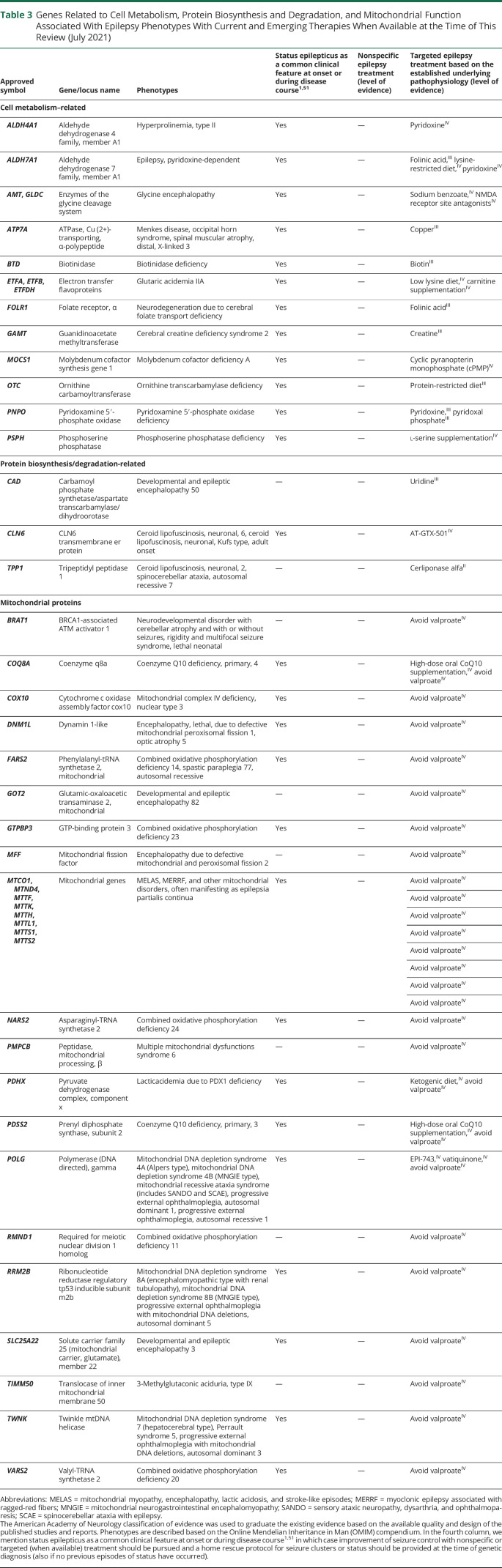
Genes Related to Cell Metabolism, Protein Biosynthesis and Degradation, and Mitochondrial Function Associated With Epilepsy Phenotypes With Current and Emerging Therapies When Available at the Time of This Review (July 2021)

### The Main Categories of Monogenic Epilepsies: From Genotype to Phenotype

#### Generalized and Focal Epilepsy Syndromes

Like other diseases with complex inheritance, a proportion of idiopathic generalized epilepsy phenotypes are caused by variants in single genes with strong effect size. For example, mutations in the *SLC2A1*, *GABRA1*, and *GABRAG2* genes account for rare sporadic or familial generalized epilepsies.

Focal epilepsies account for 50%–60% of all epilepsies and are divided into those with a structural etiology and nonacquired focal epilepsies (NAFEs). Examples of focal epilepsies with a structural genetic etiology include *COL4A1*-related porencephalic cysts and other nonspecific brain abnormalities causing focal epilepsies, *GNAQ* mosaic mutations causing Sturge-Weber syndrome, and cerebral cavernous malformations caused by mutations in *KRIT1*, *CCM2*, or *PCDC10.* Inherited or de novo germline mutations in the mammalian target of rapamycin (mTOR) pathway genes (i.e., *AKT3*, *DEPDC5*, *MTOR*, *NPRL2*, *NPRL3*, *PIK3CA*, *PIK3R2*, *TSC1*, *TSC2*) cause focal epilepsies, with or without visible brain malformations, often with neurodevelopmental disorders. Somatic mutations in mTOR pathway genes can also cause epileptogenic brain malformations such as type II focal cortical dysplasia (FCD), hemimegalencephaly, and tuberous sclerosis complex (TSC). In some individuals with an already identified germline mutation, a somatic second hit mutation in the same or different genes of the mTOR pathway has been identified in surgically removed brain tissue.^6,7^ The diagnostic yield of genetic testing in NAFE tends to be low^8^ and in many the etiology remains unknown. However, a genetic diagnosis should still be pursued as it may prompt specific management and treatment strategies. Examples of monogenic NAFEs include autosomal dominant sleep-related hypermotor epilepsy, caused by mutations in *CHRNA4*, *CHRNA2*, *CHRNB2*, *DEPDC5*, *KCNT1*, *NPRL2*, or *NPRL3*, familial focal epilepsy occasionally reported with *DEPDC5* mutations, autosomal dominant lateral temporal lobe epilepsy caused by *LGI1* mutations, and focal epilepsy with rolandic spikes caused by *GRIN2A* mutations.

#### Developmental and Epileptic Encephalopathies

Developmental or epileptic encephalopathies (DEE/EEs) are characterized by early onset of seizures, typically pharmacoresistant, often accompanied by severe epileptiform EEG discharges, both of which may affect neurodevelopment. DEE/EEs include syndromes such as early infantile epileptic encephalopathy, West syndrome, epilepsy of infancy with migrating focal seizures, Dravet syndrome (DS), and Lennox-Gastaut syndrome. “Monogenic” deleterious mutations are identified in up to ∼40% of the DEE/EEs.^2^ Identifying the underlying genetic abnormality of EE/DEEs is critical as some are potentially treatable and pharmacotherapy can be rationalized in a number of conditions. A large number of monogenic epilepsies include channelopathies, where pathogenic mutations result in a gain or loss of function of voltage-gated or ligand-gated ion channels and phenotype severity usually correlates with the degree of functional impairment of the channel involved.^9^ In addition, gain vs loss of function of the same channel can both, on occasion, result in epilepsy but usually with different phenotypes, as in *GRIN2A*-, *SCN2A*-, *SCN1A*-, and *SCN8A*-related epilepsies. Monogenic DEE/EE also include metabolic conditions (e.g., GLUT1 deficiency syndrome caused by *SLC2A1* mutations), synaptopathies (e.g., *VAMP2-*related neurodevelopmental disorders), alteration in lysosomal homeostasis (e.g., *ATP6V1A-*related DEE), cell adhesion molecules (e.g., *PCDH19*-related EE), transporters (e.g., *SLC13A5*-related DEE), secreted proteins (e.g., *SERPINI1*-related progressive myoclonic epilepsy), and alteration in neuronal proliferation, migration, and differentiation (e.g., *HECW2*-related DEE) (Tables 1–3).

### Antiseizure Treatment in the Most Prevalent Monogenic Epilepsies

The identification of a genetic etiology can lead to specific choices of ASM treatment in a small but growing number of genetic epilepsies. Treatments may include conventional ASMs or repurposed therapies (i.e., with specific actions that may have been used in entirely unrelated conditions). Treatment alteration may be guided by empirical clinical observation or targeted to the underlying specific pathophysiologic abnormality determined by the genetic etiology (Figure). We illustrate the most common monogenic childhood-onset epilepsies^1^ with their most appropriate treatment approaches.

**Figure:**
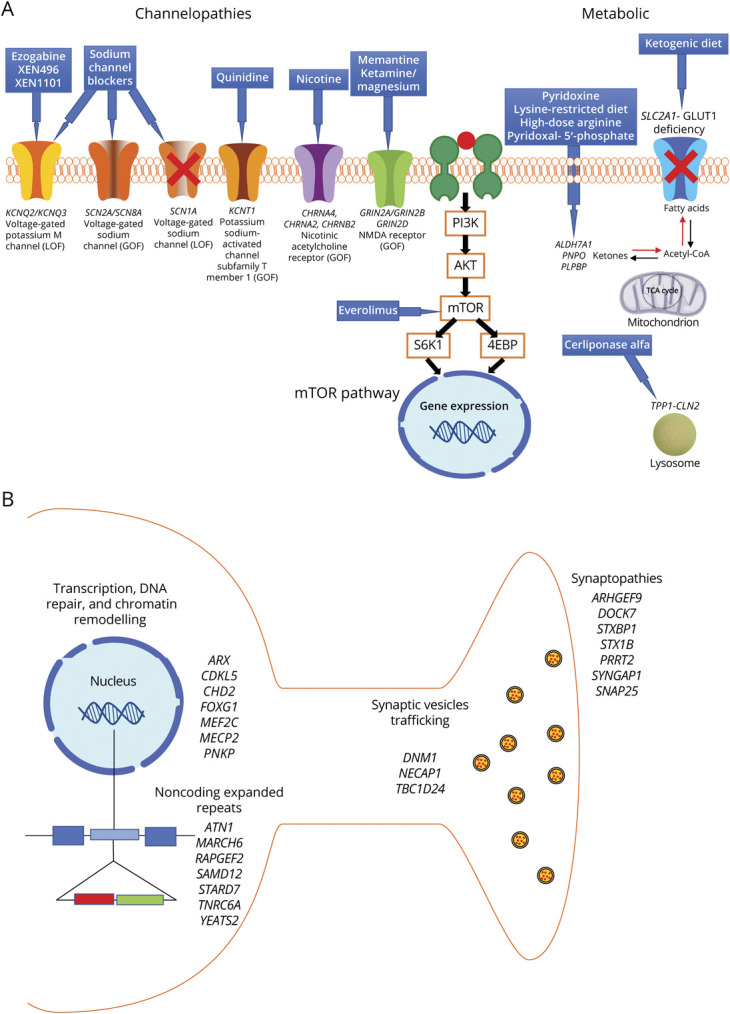
Schematic Representation of the Disease Mechanisms Associated With Genetic Epilepsies (A) Channelopathies, mTORopathies, and metabolic conditions where there is available targeted treatment based on the established underlying pathophysiology and (B) other genetic mechanisms including alteration of transcription, DNA repair, chromatin remodeling, synaptic pathways, and noncoding expanded repeats, where there are no available precision treatments.

#### *PRRT2*-Related Epilepsy

Mutations in *PRRT2* cause self-limited (familial) infantile seizures and represent the most common monogenic epilepsy with an incidence of 1 per 9,970 live births.^1^ *PRRT2*-related disorders also include paroxysmal kinesigenic dyskinesia, where sodium channel blockers are the first-choice drugs. Seizures are usually responsive to a single ASM, including carbamazepine, oxcarbazepine, valproate, or phenobarbital.^10^ *PRRT2* is thought to be involved in the modulation of synaptic neurotransmitter release, and most mutations lead to haploinsufficiency. Lack of PRRT2 leads to hyperactivity of voltage-dependent sodium channels, therefore causing alteration of neuronal excitability, and this may explain the efficacy of sodium channel blockers.^11^

#### Dravet Syndrome due to *SCN1A* Mutations

In DS, a DEE caused by loss-of-function *SCN1A* gene mutations, controlled trials have shown the efficacy of stiripentol, cannabidiol, and fenfluramine.^12-14^ There is no evidence that these drugs target specific pathophysiologic mechanisms in DS. Fenfluramine and cannabidiol are being tested in a variety of other severe epilepsies,^15,16^ while stiripentol may also be effective in other epilepsies.^17^ In a randomized, placebo-controlled trial of children with DS on stable doses of clobazam and valproic acid, 71% of those receiving add-on stiripentol vs only 5% receiving placebo achieved a >50% reduction in seizures.^12^ A 12-year prospective observational open-label study in DS showed that stiripentol improves long-term seizure frequency in approximately 50% of patients and significantly reduces the frequency of status epilepticus.^18^

Cannabidiol demonstrated efficacy in DS, with median percent reduction in target seizures of 38.9%, significantly higher than placebo treatment.^13^

After initial observations that that 70% of 12 persons with DS achieved seizure freedom with fenfluramine,^19^ 2 randomized, placebo-controlled trials were conducted. In the first, fenfluramine vs placebo was added to ASM regimens that did not contain stiripentol.^14^ Higher dose fenfluramine was associated with a significantly greater likelihood of achieving a >50% and a >75% reduction in convulsive seizure frequency. In the second trial, fenfluramine vs placebo was added to children on concurrent stiripentol and those on fenfluramine were significantly more likely to achieve both a >50% and a >75% reduction in convulsive seizures.^20^ Further active trials are assessing the efficacy, safety, and tolerability of fenfluramine and cannabidiol in children and adults with DS.^5^ Fenfluramine acts as a serotonin releasing agent but also affects the sigma receptors. There are other serotonin modulators that were shown to be effective in suppressing seizures in DS experimental models, including clemizole and lorcaserin, for both of which there are ongoing multicenter, double-blind, randomized, placebo-controlled trials in DS.^5^

Recently released but unpublished data on soticlestat, a 24-hydroxylase cholesterol enzyme inhibitor, showed that it significantly reduced convulsive seizures in DS compared with placebo. Currently there is an active phase 2, prospective, multicenter extension study to assess the safety and tolerability of soticlestat in rare epilepsy syndromes, including DS.^5^

Given the underlying *SCN1A* loss of function, sodium channel blockers should be avoided in DS.^21^

#### KCNQ2-Related Epilepsy

Early-onset DEE due to loss-of-function mutations in *KCNQ2*, encoding for the Kv7.2 voltage-dependent neuronal potassium channel subunit, responds well to sodium channel blockers and it is thought that early effective treatment may reduce cognitive disability. Voltage-gated sodium channels and *KCNQ* potassium channels colocalize and are bound at critical neuronal locations (such as the axon initial segment where action potentials are initiated and nodes of Ranvier regulate action potential propagation); blocking sodium channels can therefore redress the functional effect of loss of function of *KCNQ2*.^22^ Additional targeted treatments include ezogabine,^23^ which directly increases the opening of *KCNQ2* channels, for which there is an ongoing randomized, double-blind, placebo-controlled, multicenter study in *KCNQ2*-DEE (EPIK),^5^ and XEN1101, a small molecule that also selectively modulates the opening of *KCNQ*2/3 (KV7.2/7.3) potassium channels, for which there is an on ongoing randomized, double-blind, placebo-controlled, multicenter study in adults with focal epilepsy.^5^

#### SLC2A1-Related Epilepsy

In epilepsies caused by genetic mutations altering metabolic pathways, correction or replacement of the metabolic deficit can reverse or attenuate the pathophysiologic dysfunction. In GLUT1 deficiency syndrome, caused by heterozygous mutations in *SLC2A1*, ketogenic diet therapies are effective as they provide an alternative source, namely ketone bodies, for brain energy metabolism, thereby treating the symptoms of neuroglycopenia.^24^ Early diagnosis and initiation of the ketogenic diet are crucial to improve brain metabolism and seizure control, although the benefit on neurodevelopment remains controversial.^25^ A novel precision therapeutic option has been proposed through red blood cell exchange transfusion based on the hypothesis that red cells may have impaired glucose uptake; an active single-site proof of concept trial is in progress (early phase 1).^5^

#### CDKL5-deficiency disorder

*CDKL5* encodes for the cyclin-dependent kinase-like 5 protein, which regulates neuronal morphogenesis and synaptic function. *CDKL5* mutations cause a severe early onset DEE for which no targeted treatment exists. A randomized, placebo-controlled trial of ganaxolone, a positive allosteric modulator of the GABA_A_ receptor, has been conducted; the results have not yet been published.^5^ There is now an expanded access program for compassionate use. A phase 2 extension study to assess the long-term safety and tolerability of soticlestat is including patients with cyclin-dependent kinase-like 5 deficiency disorder.^5^ Fenfluramine is also being trialed.^5^ Short-term efficacy with ketogenic diet has been reported in a retrospective uncontrolled study.^26^

#### PCDH19-Related Epilepsy

*PCDH19* encodes for one of the cadherin superfamily of cell–cell adhesion molecules with diverse roles in neuronal migration, neuronal cell specification, or synaptic function, and its mutation causes *PCDH19* girls-clustering epilepsy (*PCDH19*-GCE). There is evidence that PCDH19 can influence GABA A receptor expression and inhibition of postsynaptic currents in the rat brain. A double-blind, placebo-controlled, phase 3 clinical study is ongoing to evaluate the efficacy and safety of adjunctive ganaxolone, a positive allosteric modulator of the GABA A receptor, in *PCDH19*-related epilepsy.^5^ A retrospective study showed that levetiracetam can be effective for seizure control in *PCDH19*-GCE, including achievement of seizure freedom in a large proportion of patients.^27^ However, the behavioral side effects of levetiracetam may limit its use in this condition, in which cognitive impairment, autistic features, obsessive-compulsive, and attention-deficit disorders are frequent comorbidities.^28^

#### SLC6A1-Related Epilepsy

*SLC6A1* encodes the GABA transporter protein type 1 (GAT1), which is one of the major GABA transporters of the human CNS. Mutations in *SLC6A1* cause neurodevelopmental disorders most likely through haploinsufficiency. There are no treatment approaches targeting the underlying pathophysiologic mechanisms. A previous observational study showed that valproate was the most effective drug, probably due to its modulation of GABA neuronal concentrations, and most patients had drug-responsive epilepsy.^29^

#### TSC1/TSC2- and DEPDC5-Related Epilepsy

The *TSC1* and *TSC2* genes causing TSC, and the *DEPDC5* gene, one of the genes most commonly associated with genetic focal epilepsy, encode for crucial inhibitory regulators of mTOR complex 1 (mTORC1), and their loss results in increased mTOR activity.^30^

Although TSC was a less frequent cause of epilepsy than *DEPDC5* mutations in the population-based study that we used as epidemiologic framework for this review, its incidence was likely underestimated as inclusion criteria envisaged epilepsy to be the clinical presentation.^1^ Adjunctive treatment with everolimus, an mTOR inhibitor, successfully reduced seizures in a randomized placebo-controlled study including patients with TSC and treatment-resistant seizures^31^ and an open-label extension phase of the study showed sustained seizure reduction over time.^32^ Hypothetical mechanisms for the antiseizure effect of mTORC1 inhibition include the inhibition of formation and growth of cortical tubers that are presumed to be epileptogenic or the reduction of inflammation. Vigabatrin is the first-line treatment for children with TSC who present with infantile spasms. Putative mechanisms to explain its efficacy include an enhanced GABA inhibitory neurotransmission and inhibition of mTOR activation.^33^ A recent multicenter clinical trial showed that vigabatrin may also be used as epilepsy-preventing treatment in TSC as it reduced the risk of clinical seizures, drug-resistant epilepsy, and infantile spasms, compared with conventional ASM initiated after the first electrographic or clinical seizure.^34^

Chronic mTORC1 inhibition with rapamycin rescues the neurologic phenotype including seizures and premature death in *DEPDC5* knockout mice^35^ and in mice with focal cortical expression of mutant mTOR.^36^ There is also experimental and clinical evidence of efficacy of the ketogenic diet in mTORopathies.^37,38^ Although the mechanisms underlying these antiseizure effects are unclear, mTOR pathway inhibition seems to contribute.^37^ There have not been dedicated medical or dietary treatment trials in patients with epilepsy caused by *DEPDC5* mutations.

### Epilepsy Surgery for Genetic Epilepsies

Surgical treatment of epilepsy can be undertaken if clinical, EEG, and imaging findings suggest focal localization of the epileptogenic zone and if any resulting neurologic deficit is not more severe than epilepsy itself. The classic principles of epileptogenic zone, ictal onset zone, and epileptogenic lesion based on surgical planning are more recently facing the challenging evidence that medically intractable focal epilepsies may be related to either germline or somatic gene mutations that may confer epileptogenicity to brain areas outside the targeted epileptogenic zone. This risk is particularly high in patients with more than one cortical lesion. In spite of these difficulties, it is recommended that epilepsy surgery be considered promptly in medically refractory TSC after failure of 2 ASMs, even in patients with multiple cortical lesions, multifocal interictal foci, and different types of seizures.^39^ Cumulative evidence indicates that patients with TSC have a 50%–60% chance of long-term seizure freedom after surgery for epilepsy, including those with infantile spasms.^40^

Germline or low-allele frequency somatic mutations in mTOR pathway genes can be demonstrated in up to 63% of patients with FCD type II,^41^ which bears close histopathologic similarities with the tubers of TSC. Somatic *mTOR* mutations and double mutational hits combining a germline and a somatic mutation of the *DEPDC5* gene are most frequently found in FCD II^36,41-43^ but the overall number of patients described is too limited to draw any conclusion on the prognostic value specific mutations in each of these genes may have. In spite of these limitations, it appears that germline mutations of *DEPDC5*, *PTEN*, *PIK3CA*, *AKT3*, *RHEB*, and *NPRL2* do not in themselves contraindicate resective surgery if a focal dysplastic lesion is present.^41,44,45^ Evidence begins to emerge that both mutational load and dysmorphic neuron density correlate with the epileptogenic zone^42^ and that mTOR mutations with strong hyperactivating properties may carry a higher risk of relapse of seizures after surgery.^46^ Although identification, quantification, and functional characterization of specific mosaic mutations could until recently be gathered only after surgery, access to cell-free DNA derived from the CSF^47^ can now demonstrate mosaic mutations before surgery. This type of information will likely become instrumental for better planning of surgical strategies and reintervention after a first unsuccessful surgery.

Experiences of resective epilepsy surgery in patients carrying germline mutations of other epilepsy genes, including sodium channel genes,^48-50^ or rare copy number variants^45^ have been rather limited but mostly disappointing, even when an MRI-visible lesion could be targeted.^48^

### Other Targets of Management

#### Avoiding Seizure Precipitants

Environmental factors may exacerbate seizures in many epilepsy types. In DS and *PCDH19*-associated epilepsy, fever or hyperthermia are important triggers and in DS, photic stimulation, music, or diaper changing, as well as fatigue or excitement, may trigger seizures. Systematic screening for specific triggers known to cause reflex seizures in genetic epilepsies should be considered to inform management strategies.

Certain ASMs may lead to seizure exacerbation. In DS, sodium channel agents such as lamotrigine, oxcarbazepine, carbamazepine, as well as vigabatrin and tiagabine should be avoided (Table 1).

#### Treatment of Acute Seizures and Status Epilepticus

In some genetic epilepsies, there is an increased risk of prolonged seizures or status epilepticus, which represent a recurrent or even initial clinical manifestation of the syndrome (Tables 1–3).^1,51^ In these conditions, early and appropriate treatment of the epilepsy may reduce the incidence of status epilepticus.^51^ In addition, home rescue therapy should be considered and the adoption of a seizure rescue protocol is recommended, as this can improve outcome by reducing mortality and morbidity. Rescue therapy should be individualized to each patient. For example, young children with DS with a history of recurrent status are often provided rescue at the onset of a convulsive seizure. Although prehospital administration of benzodiazepines may be effective to control seizure clusters or status epilepticus and avoid hospital admission, excessive doses should be avoided due to higher risk of respiratory depression and longer hospitalization.^52^ The spectrum of action of drugs used in status epilepticus may differ from the epilepsy. For example, although phenytoin is contraindicated in DS, it has been used successfully against status epilepticus in some patients.^53^

### Gene Therapies

There have been considerable recent advances in the development of therapies to target genetic disease. The introduction of new genetic material, modification of the genome, and modification of DNA transcription all fall under the rubric of “gene therapy.” The growth in gene therapies in medicine has partly been realized through the development of safe and effective means of gene delivery using viral vectors. Viral vectors are viruses that have had the genetic instructions for replication removed and replaced with a desired cargo, which consists of a promoter (determining the cell type in which the gene will be expressed) and the target gene. This approach has certain drawbacks. Many of our present viral vectors can only carry a limited amount of DNA, restricting the size of the genes that can be expressed, and most vectors have to be injected into a restricted area of brain. However, improvements in vector design have permitted widespread expression throughout the brain with intraventricular injections and also peripheral injection of vectors that can cross the blood–brain barrier, enabling use in genetic epilepsies.^54^

Gene therapy treatment approaches can have the advantage of specifically targeting the mutated gene or the consequent protein expression. However, simply introducing unexpressed or underexpressed genes can be problematic. Some genes, such as *SCN1A* for DS, are too large for our present vectors. Also, there is little way to control “dosing” and there is therefore the risk of overdosing a gene, which could have a detrimental effect on cell viability or excitability.

An alternative approach to introduce a gene is gene editing. One of the simplest and most widely used gene editing tools is the CRISPR-Cas9 system. This makes use of a guide RNA that directs the enzyme Cas9 to a specific part of the genome where it cuts the DNA. This ever-increasingly sophisticated approach can repair or knockout genes, but its clinical translation has been hampered by varying efficiency, off-target effects, and, on occasion, insufficient vector size for the necessary genetic material. From a translational perspective, the dCas9 system may have greater traction. Here Cas9 is mutated to “dead” Cas9, which no longer cuts DNA and is instead fused with gene transcription regulators either activating or repressing genes neighboring the guide RNA binding site. The advantage of this system is that the genetic material can be easily contained in an adeno-associated viral vector. This approach has been used in a mouse model of DS to upregulate Scn1a expression in interneurons using a Scn1a-dCas9 activation system in an adeno-associated viral vector.^55,56^ This rescued interneuronal excitability, behavior, and attenuated hyperthermic seizures. The disadvantage of the CRISPR-Cas9 system is that Cas9 is a foreign protein and so potentially immunogenic.

Although targeting the abnormal gene or gene products would seem the most obvious strategy, it may not always be the best. This can be because gene mutations often have a developmental effect and so reversing the genetic cause postnatally may not reverse the effect of that mutation. Moreover, it may be possible to successfully treat the seizures but without an effect on the comorbidities. This may apply not only to developmental genes, but also to receptors and channels, some of which can modify brain development. Moreover, a mutation in one gene may have an effect on the expression of multiple other genes, which can then contribute to the development of epilepsy. An example is the mTOR-dependent expression of Kv1.1.^57^ The mTORopathies have decreased levels of Kv1.1 channel expression; because Kv1.1 is a powerful regulator of neuronal excitability and has potent antiseizure effects,^58^ upregulating Kv1.1 provides an alternative and a potentially more attractive and more easily translatable strategy to treat the mTORopathies.

An alternative to targeting DNA is to target transcription. mRNA transcription can be regulated using an antisense oligonucleotide (ASO), a single-stranded deoxyribonucleotide, which is complementary to the mRNA target, or double-stranded RNA-mediated interference. The latter is an endogenous system used by cells to regulate gene expression through noncoding small sequences of RNA (small interfering RNA [siRNA]) binding to mRNA, resulting in mRNA degradation. siRNA can be injected directly into the brain/CSF or can be coupled with cell penetrating peptides to enable peripheral administration and penetration across the blood–brain barrier. ASOs are usually administered intrathecally. ASO binding to mRNA can have several different possible effects: it can inhibit mRNA transcription, alter mRNA splicing, or increase mRNA degradation. Intraventricular administration of an ASO directed against the Scn8a transcript in a mouse model of an *SCN8A* gain-of-function mutation–associated encephalopathy delayed seizure onset and increased survival.^59^ Following transcription of DNA to produce precursor mRNA, the precursor mRNA is spliced to remove introns and join together exons. This splicing can occur multiple ways, so that most mammalian genes generate multiple mRNA versions (splice variants). Many of these splice variants are not translated and degrade. Because ASOs can regulate splicing, it is possible to use ASOs to increase the production of translated mRNA. This has been termed targeted augmentation of nuclear gene output (TANGO). TANGO has recently been used in an animal model of DS, increasing Scn1a transcript and the production of the sodium channels, reducing seizures and sudden death in epilepsy.^60^ This approach is undergoing a phase 1 and 2 clinical trial in DS.^5^

Lastly, a less precise but simpler approach to redress point mutations that prematurely terminate mRNA, and consequently prevent full-length protein expression, has been the emergence of small molecules that induce translational read-through, suppressing stop codons and consequently resulting in the synthesis of full-length proteins. Ataluren is one such drug that is undergoing a trial in a small number of people with nonsense mutation DS or CDKL5 deficiency.^5^

Clinicians who manage patients with genetic epilepsies should be aware of the implications that specific genetic etiologies may have on pathophysiology, natural history, and the associated comorbidities. The choice of ASMs for these patients is informed by a growing body of knowledge derived from completed and ongoing trials on patients with specific disorders and inferences drawn from observational studies and initial experiences with precision therapies. Despite the lack of robust evidence for most genetic epilepsies, examples of successful precision medicine application are increasing and new methodologies for treatment trials are emerging, such as N-of-1 trials. Ongoing and completed clinical trials for genetic epilepsies can be found online on the clinicaltrials.gov website.^5^

Although correlations between highly penetrant mutations are easier to grasp and may be seen as elective targets for precision treatment approaches, caution is required in inferring that pathophysiologic mechanisms can be reversed or antagonized after identifying a mutation in a given gene. Phenotypic heterogeneity is remarkable, and some genetic mutations activate epileptogenesis through their developmental effects, which may not be reversed postnatally, while other genes having seemingly pure functional consequences on excitability may act through either loss- or gain-of-function effects, and these may have opposite treatment implications. In addition, the functional consequences of missense mutations may be difficult to predict, making precision treatment approaches considerably more complex than estimated by deterministic interpretations.

## Glossary

ASM: antiseizure medication
ASO: antisense oligonucleotide
DEE: developmental epileptic encephalopathy
DS: Dravet syndrome
EE: epileptic encephalopathy
FCD: focal cortical dysplasia
mTOR: mammalian target of rapamycin
mTORC1: mammalian target of rapamycin complex 1
NAFE: nonacquired focal epilepsy
*PCDH19*-GCE: *PCDH19* girls-clustering epilepsy
siRNA: small interfering RNA
TANGO: targeted augmentation of nuclear gene output
TSC: tuberous sclerosis complex
